# The effect of moderate and vigorous aerobic exercise training on the cognitive and walking ability among stroke patients during different periods: A systematic review and meta-analysis

**DOI:** 10.1371/journal.pone.0298339

**Published:** 2024-02-23

**Authors:** Zecheng Li, Hongpeng Guo, Yuan Yuan, Xuebin Liu

**Affiliations:** 1 College of Sports Science, Harbin Normal University, Harbin, China; 2 College of Medical Information Engineering, Shandong First Medical University, Tai’an, China; Iran University of Medical Sciences, ISLAMIC REPUBLIC OF IRAN

## Abstract

**Objective:**

The study examined whether rehabilitation using aerobic exercise is more appropriate for patients less than 3 months post-stroke or more appropriate for patients more than 3 months post-stroke.

**Method:**

PubMed, Embase, Web of Science, Scopus and CNKI databases were searched from inception to September 2023. All studies included must be written in English and grey literature was excluded. The quality of the study was evaluated using the PEDro scale. Standard mean difference (SMD) and 95% confidence interval (CI) were calculated. The primary outcomes are cognitive ability and walking ability. The intervention of the experimental group must be or include high-intensity aerobic training or moderate-intensity aerobic training. In addition, we required low intensity routine exercises in control group.

**Result:**

Only 15 studies were included in this meta-analysis. The results showed that aerobic exercise has a positive rehabilitation effect on cognitive and walking ability of stroke patients. Global Cognitive Function (SMD = 0.81 95%CI 0.49–1.12), Walking Capacity (SMD = 1.19, 95%CI 0.75–1.62), VO_2_peak (SMD = 0.97, 95%CI 0.66–1.28), and brain-derived neurotrophic factor (SMD = 2.73, 95%CI 2.03–3.43). We further observed that patients who suffered a stroke within the past three months exhibited superior rehabilitation outcomes compared to patients who suffered a stroke more than three months ago, specifically in terms of cognitive ability, walking tests, and cardiopulmonary function.

**Conclusions:**

It is recommended to carry out treatment for patients in the initial stage of stroke, and it is required to pay attention to exercise intensity in the process of treatment to ensure patient safety.

## Introduction

Stroke is a common threat to mental and physical disability, which posed high burden on mortality and disability rate [1], with the incidence of stroke increasing from 1.1 million / year in 2000 to 1.5 million / year in 2025 [2, 3]. According to studies, stroke patients have a decline in their ability to live independently. Stroke causes serious damage to the patient’s cardiovascular function and brain. Moreover, in the United States, the risk of stroke has increased by 25% since 2010. More than 4 million people will be sick in 2030 predicted by previous studies [4]. Therefore, it is very important to explore effective treatment methods for stroke patients to improve their cognitive and living ability.

Previous studies have shown that appropriate physical exercise and physical activity are effective means for stroke patients to restore physical function [4–6]. Many researchers have carried out a lot of research on exercise interventions suitable for stroke patients [7, 8], the vast majority of experimental results show that the therapeutic effect of aerobic exercise is the most significant [9]. Compared with traditional rehabilitation training methods, aerobic exercise has more obvious stimulation on cardiopulmonary function and brain cognition, which can enhance the cognitive ability of stroke patients [10], reduce pain [11], relieve cardiovascular pressure [12], and improve walking ability [13, 14].

Although the use of aerobic exercise in post-stroke treatment and rehabilitation has been comprehensively explored, it is unclear how aerobic exercise differs in its therapeutic effects on post-stroke patients at different periods of rehabilitation. Due to the decline in cardiovascular and cerebrovascular function after stroke, there are differences in the level of physical function of stroke patients at different times. Therefore, when performing aerobic exercise interventions, therapists need to choose the appropriate exercise intensity according to the differences in stroke duration in stroke patients. In conclusion, what kind of stroke patients are most suitable for aerobic exercise therapy, and whether aerobic exercise is more suitable for patients with a shorter post-stroke duration or patients with a longer post-stroke duration are the main questions of this review.

Therefore, to address the gaps, we conducted a comprehensive meta-analysis to divide patients into two subgroups of stroke time greater than 3 months and less than 3 months. On this basis, this study aims to provide exercise recommendations and programs suitable for the rehabilitation of patients with different stroke periods.

## Method

### Search strategy

This meta-analysis followed the Preferred Reporting Items for Systematic Reviews and Meta-Analyses guidelines (PRISMA) and the Cochrane Handbook for Systematic Reviews of Interventions. The review protocol was registered with PROPERO (CRD42023463751).

Only randomized control trial (RCT) investigations were enrolled in this meta-analysis, and this research was conducted with Review Manager Software (RevMan5.4), using the PubMed, Embase, Web of Science, Scopus and CNKI databases, which were searched from inception to September 2023. Based on each database, different combinations of possible keywords and Medical Subject Headings terms were used. All studies included in the study must be written in English grey literature were excluded. We also checked the references of the included articles to find any missing article. The search strategy was shown in S1 Table.

### Screening process

We followed the PICO standard to develop strict inclusion criteria. All RCTs must meet the inclusion criteria to be considered in this study. The title and abstract of the articles are reviewed by two authors respectively and the final decision is made by the third author. The studies meeting the criteria were moved to the subsequent full-text review. After the screening of the article, we still used two authors to screen, and the third author finally decided to judge the follow-up work such as literature quality assessment and risk assessment.

### Eligibility criteria

For the participants, we required that all participants of the included studies be patients with hemorrhagic and ischemic stroke. According to the design of this meta-analysis, we required all patients to be sick for more than 3 months or less than 3 months, and the age must be more than 18 years. All the patients included in the study had no drug use records before and after the exercise intervention, so the influence of factors such as drugs on the results of the article can be excluded.

For the intervention, Li [15], Mah and Amoros-Aguilar [16, 17] proposed in the study that moderate and vigorous aerobic training has the best rehabilitation effect on stroke patients. And there is little difference between the therapeutic effect of moderate intensity aerobic exercise and the therapeutic effect of high intensity aerobic exercise. Hence, this study aims to set moderate and vigorous aerobic training as the intervention measure of the study. The intervention of the experimental group must be or include high-intensity aerobic training or moderate-intensity aerobic training. About the control group, we required the low intensity routine exercises. The routine exercise program consisted of stretching exercise, balance training, functional exercise, cognitive training and usual care with medicine.

The studies included must contain one or more of the following outcome indicators, otherwise it will be excluded.

The primary outcomes are cognitive ability and walking ability. We used the Mini-Mental State Examination score (MMSE) and Montreal Cognitive Assessment (MoCA) to measure the improvement of training effect on global cognitive function. In the study of cognitive function, Li [15] integrated MoCA, MMES and Addenbrooke’s cognitive examination revised (ACE-R) as a measurement tool to evaluate cognitive function, and showed its feasibility. Therefore, although there are differences in the cognitive domains measured by MoCA and MMES, they are all data tables for measuring cognitive level in the final analysis, and have been practiced by scholars. Therefore, in this study, we also used the results of MoCA and MMES as the basis for cognitive function tests. Then we used 6-minute walk test (6WMT) to evaluate walking capacity.

The secondary outcomes are brain-derived neurotrophic factor (BNDF) changes and VO_2_peak. It is well known that BNDF played a vital role in the development of nervous system cells such as glial cells, neuronal cells, hippocampus and cortical neurons [18]. In addition, some studies found that the cardiovascular health level of stroke patients can be expressed by peak oxygen uptake (VO_2_peak) [19, 20]. So, we evaluated the improvement effect of exercise therapy on some physiological indexes of stroke patients by BNDF and peak oxygen uptake (VO_2_peak).

### Data extraction

For study selection and data extraction, we followed the predetermined criteria to search related studies The following data were extracted from the included studies: information of participants (age, gender, numbers), intervention design (experimental and control), treatment condition (frequency and duration), outcome and drop-out. In addition, we created a table using this data (see S2 Table).

### Risk-of-bias assessment

To assist the risk of bias, we evaluated the risk of bias for each included studies using the Cochrane Handbook for Systematic Reviews of Interventions [21] and PEDro scale [22]. A third reviewer will be involved if two reviewers dispute the results.

For the publication bias, although the number of articles included in this article is more than 10, the publication bias analysis cannot be performed through the funnel plot as the number of articles in the subgroup is less than 10.

### Statistical analysis

For statistical analysis, we used Review Manager Software (RevMan5.4) to analyze the post-intervention results of the studies we included. We chose the standardized mean difference (SMD) as a useful sign, and provided a 95% confidence interval (95% CI) for the variance. Heterogeneity was assessed using the Q statistic and the I^2^ test, where P < 0.05 (2-tailed) or I^2^ values greater than 50% were considered significant. Since our data is random, we chose the random-effects (RE) model.

## Result

### Search and screening results

**Fig 1:**
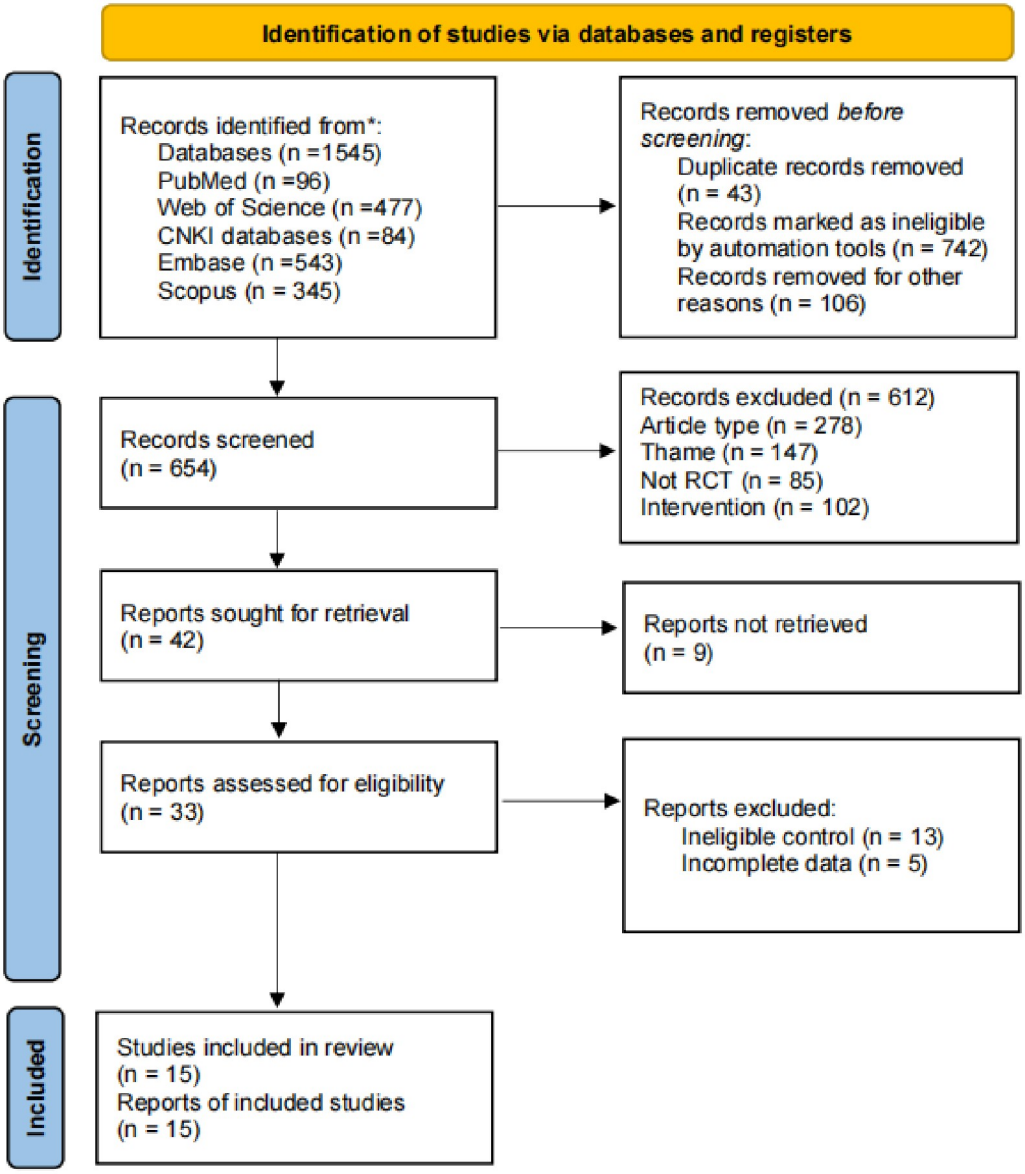
PRISMA flow chart.

We retrieved a total of 1545 articles, after which we removed 742 and 43 duplicates through automated tools, then 612 articles were excluded following the inclusion criteria. Finally, a total of 15 studies were included in this meta-analysis. Among them, the number of stroke patients greater than 3 months is more than that of stroke patients less than 3 months, and the age of the patients is concentrated in the 50–70 years old, which belongs to the middle-aged and elderly patients. The complete PRISMA flow chart is shown in Fig 1.

### Study characteristics

A total of 15 articles were included in this study [23–36], of which 7 articles involved indicators related to cognitive ability (MMSE and MoCA), 8 articles applied 6WMT, 3 articles studied the numerical changes of BNDF and 6 articles measured the changes of VO_2_peak after exercise intervention.

In addition, Lapointe [31] set up two experimental intervention groups of moderate-intensity aerobic and high-intensity aerobic in the study. Liu [35] also set up two experimental groups according to the patient ’s stroke time in the study: stroke more than 3 months and stroke less than 3 months. The study of this meta-analysis involved the effect of moderate and high intensity aerobic exercise on stroke patients, and the rehabilitation effect of aerobic exercise on patients with stroke for more than 3 months and patients with stroke for less than 3 months were also studied. Therefore, we decided to record the two sets of experimental results of Lapointe and Liu as Lapointe (a), Lapointe (b), Liu (a), Liu (b), and included them in the study. See S2 Table for details.

### Quality assessment and risk of bias

**Fig 2:**
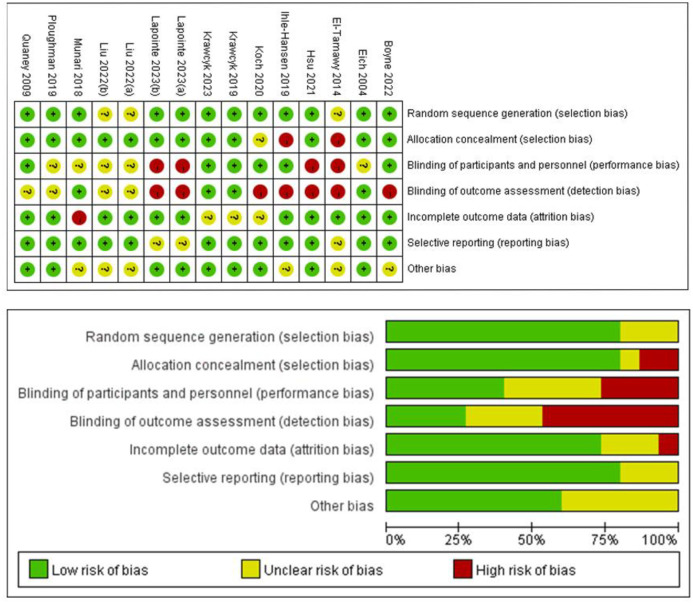
The result of risk of bias.

We used the Cochrane Handbook for Systematic Reviews of Interventions and PEDro scale to evaluate the quality of inclusion studies. The detailed evaluated results are shown in S3 Table and Fig 2. Generally, the overall quality level of the literature is relatively high. The PEDro score of all studies were higher than 6 points, and the PEDro score of 3 studies were 10 points. Bias assessment shows that, only selection, performance, detection and attrition have a higher risk, and the other parts have no apparent risk of bias.

### Outcomes of interest

#### Exercise strategy

From the basic characteristics of the table included in the study, we found that most of the experimental interventions are aerobic exercise that will stimulate the patient ’s heart rate to a certain extent, and the exercise intervention time is not long, generally between 45–60 min.

In terms of intensity, although there are differences in the assessment methods used to determine the intensity of exercise, they are all based on the patient ’s heart rate. Additionally, most of the studies were based on 60% of the maximum heart rate as the average intervention intensity, only a small number of studies reached 80%–90% of the maximum heart rate intensity. See S2 Table for details.

#### Global cognitive function

We used a random forest plot to display the results of data analysis, and divided the results into two subgroups according to the stroke time of patients: stroke time of more than 3 months and stroke time of less than 3 months.

**Fig 3:**
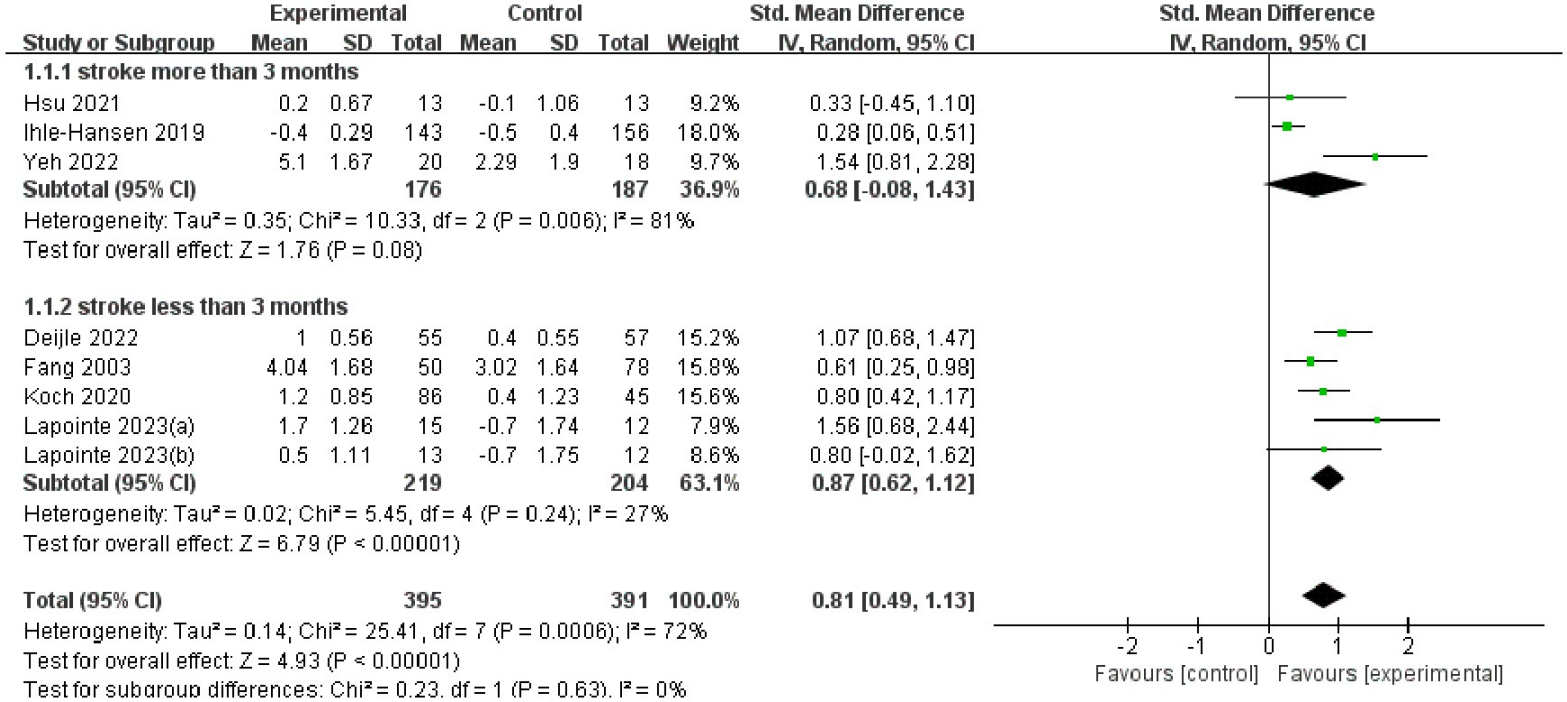
The forest plot of global cognitive function.

The overall heterogeneity of the results after analysis was 72%, and the heterogeneity between subgroups was 0%. The improvement effect of patients with stroke time less than 3 months (SMD = 0.87, 95%CI 0.62–1.12) is proved to be higher than that of patients with stroke time more than 3 months (SMD = 0.68, 95%CI -0.08–1.43). See Fig 3 for all details.

#### Walking capacity

**Fig 4:**
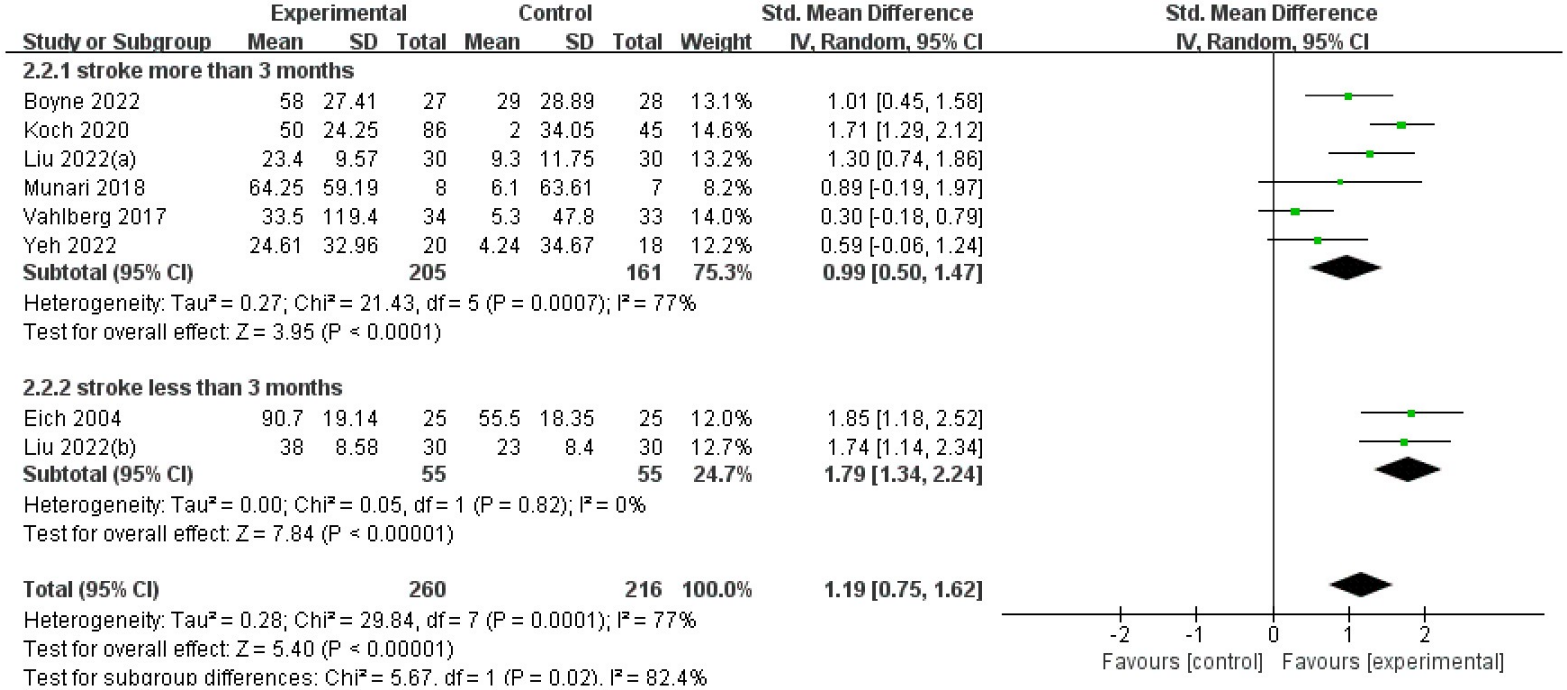
The forest plot of 6WMT.

We used 6WMT as an indicator to measure walking capacity, madding a random forest plot and performing sensitivity analysis on the results. The results of the experimental group are better than the control group (SMD = 1.19, 95%CI 0.75–1.62), less than 3 months of stroke patients treatment effect (SMD = 1.34, 95%CI 1.34–2.24) is better than more than 3 months of stroke patients (SMD = 0.99, 95%CI 0.50–1.47). See Fig 4 for all details.

#### VO_2_peak changes.

**Fig 5:**
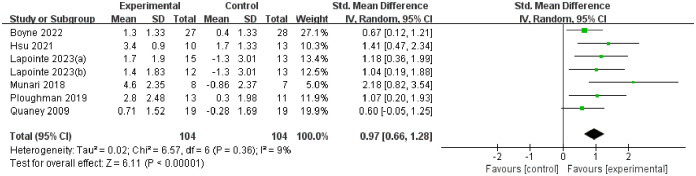
The forest plot of VO_2_peak changes.

The overall heterogeneity of the results of VO_2_peak was very low (P < 0.00001, I^2^ = 9%), and overall results showed that the experimental group had a positive improvement effect on stroke patients (SMD = 0.97, 95%CI 0.66–1.28). See Fig 5 for all details.

#### BNDF changes

**Fig 6:**
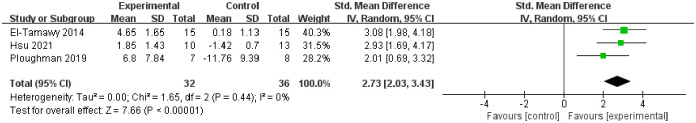
The forest plot of BNDF changes.

Three studies measured the changes of BNDF. The analysis results showed that moderate and high intensity aerobic exercise promoted the increase of BNDF content in stroke patients (SMD = 2.73, 95%CI 2.03–3.43), Moreover the experimental results were significant and the heterogeneity was low (P<0.0001, I^2^ = 0%). See Fig 6 for all details.

## Discussion

The results of this meta- analysis show that aerobic exercise has a positive rehabilitation effect on the cognitive and walking ability of stroke patients. After moderate or high-intensity exercise intervention, the exercise ability, cardiovascular health level and cognitive ability of stroke patients have been improved. Subjected to the limited number of the included studies, it is hard to ensure the similarity in the number of two subgroups when classifying subgroups, and it is difficult to make sure that the include studies were used unified training methods, the results are statistically heterogeneous.

### Previous studies and results discussion

The results of this study on cognition, exercise capacity, cardiovascular function and brain neurotrophic factors are consistent with those of other scholars [8, 13, 14]. Our results show that appropriate walking training has a favorable effect on the rehabilitation of walking ability, which is consistent with the results of Pang [14] on the improvement of physical health of stroke patients by lower limb exercise. In addition, our results also echo the results of Anjos study on the improvement of walking speed in patients with aerobic exercise, and the conclusion that aerobic exercise promotes cardiopulmonary function in stroke patient [13]. Although there is a certain heterogeneity in the results of cognitive improvement, it has a positive effect on the improvement of patients ’ cognitive ability, which is similar to the results of Li [15] and Tiozzo [8].

In terms of cognitive function, we believe that combining the results of MoCA and MMSE and evaluating them will not affect the experimental results, but will lead to their heterogeneity. In addition, we found that the content of BNDF increased significantly after the experiment, and brain neurotrophic factors played an essential role in improving cognitive ability and brain development. Because exercise could promote the increase of BNDF content [37], we also believe that part of the reason for improving the cognitive ability in the experimental results lie in the exercise stimulation to the brain to produce more brain neurotrophic factors.

Walking endurance is a part of walking ability. It is an important index to evaluate the rehabilitation degree of stroke patients [38]. From the 6WMT results, aerobic exercise significantly improved the walking endurance of stroke patients (SMD = 1.19), but the heterogeneity is high. In order to further explore the source of heterogeneity, we re-examined research articles involving 6WMT. We found that the exercise methods of aerobic exercise intervention in the experimental group were not uniform. Most studies use treadmill exercise or aerobic cycling for aerobic exercise, but some studies have achieved the effect of aerobic exercise through strength training and resistance exercises. We believe that there are some differences in the degree of stimulation of patients with different exercise methods. Because 6WMT is a test of lower limb motor ability, the study group that takes treadmill exercise and bicycle training to exercise the lower limbs in the process of aerobic intervention should be superior to other exercise groups in the outcome indicators, which is the main reason for the high heterogeneity of 6WMT results.

As this paper primarily focuses on enhancing cognitive and walking capabilities, without delving into mitigating the risks of cardiovascular dysfunction, the literature incorporated herein does not concentrate on assessing cardiovascular function enhancement. Consequently, subgroup analyses of VO2peak outcomes are not performed. The findings reveal that aerobic exercise yields substantial and noteworthy benefits in boosting oxygen uptake for stroke patients.

### Meaning and uniqueness

The most significant difference between this study and existing studies is that subgroup analysis is performed according to the patient ’s stroke time. Therefore, when discussing the results, we are more inclined to explore how to formulate corresponding exercise strategies according to the sick time of stroke patients. Pang has proposed different intensity of exercise therapy for the personality characteristics of stroke patients in previous studies. However, it is only a simple analysis which does not explore the relationship between the rehabilitation effect of aerobic exercise and the stroke time of patients [14].

From a deepening perspective, we explore the rehabilitation effect of aerobic exercise. The results showed that the rehabilitation effect of patients with stroke less than 3 months was better than that of patients with stroke more than 3 months in terms of cognitive ability, walking test and cardiopulmonary function. The results highlighted that exercise therapy is more suitable for the rehabilitation training of patients with early stroke. The reason was the brain spontaneously repairs damaged nerve connections within a certain period in the acute phase of stroke [39–41]. Over time, in the chronic phase of stroke, both the brain ’s neuroplasticity and the individual ’s ability to repair itself in the event of injury has been reduced [42].

### Suggestions of exercise strategy

The early stage after stroke is a dynamic and changeable. Exercise intervention for early patients should fully consider the changes in all physiological systems and detect the adverse physiological reactions of patients in time. Great importance should be attached to the exercise therapy for the degree of elevated blood pressure during exercise and the consequent exercise hypotension [43], so the exercise strategy for patients with early stroke should be based on safety and reduce exercise intensity. When the exercise intensity reaches 60% -70% of the maximum oxygen uptake, the cerebral blood vessels will contract and make the cerebral blood flow tend to rest or decrease, thus playing a protective role in the brain. However, due to damage to the brain of stroke patients, it is often challenging to cause cerebrovascular contraction [44], resulting in higher risks in high-intensity exercise facing stroke patients.

Hence, it is crucial to regulate exercise intensity during exercise intervention. When engaging in moderate to high-intensity exercise, it is essential to maintain a heart rate between 120–140 beats per minute, while keeping the duration of each session within a reasonable limit. For patients who have suffered a stroke within the past three months, the duration of each exercise session should not exceed 30 minutes. For patients who have suffered a stroke for more than three months, the intensity and duration of exercise can be gradually increased, but should not exceed a maximum of 60 minutes per session.

### Limitations

We analyzed the health indicators of cognition, exercise ability, cardiovascular and other aspects in order to explore the effect of moderate and high intensity aerobic exercise on the health promotion of stroke patients. Some limitations are important to note for this study. For cognitive function, we did not study the effect of executive function improvement, and did not evaluate memory and response ability. For cardiovascular function, there is still a gap in cardiac health promotion. In addition, this paper does not involve balance ability detection and gait analysis for daily walking ability. Therefore, we can only preliminarily prove that moderate and high-intensity aerobic exercise has a certain self-care ability improvement and health promotion function for stroke patients, which can be further studied in future research.

Moreover, the number of literatures included was restricted to 15. After subgroup analysis according to the patient ’s stroke time, there were only 2–3 literature data in some subgroups. Therefore, even if our study shows that exercise intervention is more effective for patients with early stroke, there is still a risk of bias, which can be supplemented in future studies and verified by more experimental data.

## Conclusions

We concluded that moderate and high-intensity aerobic exercise is conducive to improving the cognitive ability and walking ability of stroke patients. Additionally, treating stroke patients in the early stages has a more effective outcome, which can improve their cognitive, walking, and cardiopulmonary functions. This can enhance patients’ aerobic potential energy and independent living ability of patients while reducing the risk of illness and morbidity. Therefore, it is recommended to provide aerobic exercise as treatment for related patients in the initial stages of stroke, and therapists need to focus on ensuring patient safety during the treatment process by monitoring exercise intensity.

## Supporting information

S1 Table Search strategy. (DOCX)

S2 Table The character of included studies. (DOCX)

S3 Table The result of PEDro scale. (DOCX)

S1 Checklist PRISMA 2020 checklist. (DOCX)
