# Supplementary material for: The effect of moderate and vigorous aerobic exercise training on the cognitive and walking ability among stroke patients during different periods: A systematic review and meta-analysis
Source: PLoS One. 2024 Feb 23;19(2):e0298339. doi: 10.1371/journal.pone.0298339 (PMC10889575; doi:10.1371/journal.pone.0298339)
Supplement: S1 Table — (DOCX) [file pone.0298339.s001.docx]

**Table S1. Search strategy**

|  | **Search term** |
| --- | --- |
| #1 | (Strokes [MeSH Terms]) OR (Cerebrovascular Accident [Title/Abstract] OR Cerebrovascular Accidents [Title/Abstract] OR Cerebrovascular Apoplexy [Title/Abstract] OR Brain Vascular Accident [Title/Abstract] OR Brain Vascular Accidents [Title/Abstract] OR Cerebrovascular Stroke [Title/Abstract] OR Cerebrovascular Strokes [Title/Abstract] OR Apoplexy [Title/Abstract] OR Cerebral Stroke [Title/Abstract] OR Cerebral Strokes [Title/Abstract] OR Acute Stroke [Title/Abstract] OR Acute Strokes [Title/Abstract] OR Acute Cerebrovascular Accident [Title/Abstract] OR Acute Cerebrovascular Accidents [Title/Abstract]) |
| #2 | (Exercises [MeSH Terms]) OR (Physical Activity [Title/Abstract] OR Activities, Physical [Title/Abstract] OR Activity, Physical [Title/Abstract] OR Physical Activities [Title/Abstract] OR Physical Exercise [Title/Abstract] OR Physical Exercises [Title/Abstract] OR Aerobic Exercise [Title/Abstract] OR Aerobic Exercises [Title/Abstract] OR Aerobic Exercise Training [Title/Abstract] OR Exercise Trainings [Title/Abstract]) |
| #3 | (Cognitive Behavioral Therapies[MeSH Terms]) OR (Therapy, Cognitive Behavioral[Title/Abstract] OR Psychotherapy, Cognitive[Title/Abstract] OR Cognitive Therapies[Title/Abstract] OR Cognitive Therapy[Title/Abstract] OR Cognitive Psychotherapy[Title/Abstract] OR Cognitive Psychotherapies[Title/Abstract] OR Cognition Therapy[Title/Abstract] OR Cognition Therapies[Title/Abstract] OR Therapy, Cognitive Behavior[Title/Abstract] OR Cognitive Behavior Therapies[Title/Abstract] OR Cognitive Behavior Therapy[Title/Abstract]) |
| #4 | (Physical Performances [MeSH Terms]) OR (Functional Performances, Physical [Title/Abstract] OR Performance, Physical Functional [Title/Abstract] OR Performances, Physical Functional [Title/Abstract] OR Physical Functional Performances [Title/Abstract] OR Physical Performance [Title/Abstract] OR Performance, Physical [Title/Abstract] OR Performances, Physical [Title/Abstract]) |
| #5 | RCT[Title/Abstract] OR randomized controlled trial [Title/Abstract] OR randomized controlled trials [Title/Abstract] |
| #6 | #1 AND #2 AND #5 |
| #7 | ((#6 AND #3) OR (#6 AND #4)) |
