# Supplementary material for: The effect of moderate and vigorous aerobic exercise training on the cognitive and walking ability among stroke patients during different periods: A systematic review and meta-analysis
Source: PLoS One. 2024 Feb 23;19(2):e0298339. doi: 10.1371/journal.pone.0298339 (PMC10889575; doi:10.1371/journal.pone.0298339)
Supplement: S2 Table — (DOCX) [file pone.0298339.s002.docx]

**Table S2. The Character of Included Studies**

| **Included studies** | **Mean age**  **(years)** | **Patient types** | **Participants (M/F)** | **Sample Size (T/C)** | **Intervention** | | **Frequency** | **Duration** | **Intensity** | **Outcome Measure** | **Drop out**  **(T/C)** |
| --- | --- | --- | --- | --- | --- | --- | --- | --- | --- | --- | --- |
|  |  |  |  |  | **Experimental** | **Control** |  |  |  |  |  |
| Boyne 2022 | T: 63.8 ± 9.9  C: 61.5 ± 9.9 | stork＞3 months | 36/19 | 27/28 | HIIT | MAT | 45 min/times  3 times/week | 12 weeks | 60% HRR | 6WMT  VO2 | 8/5 |
| Deijle 2022 | T: 64.7 ± 8.9  C: 63.9 ± 10.6 | stork＜3 months | 70/49 | 60/59 | HAT | Routine exercises | 60 min/times  2 times/week | 12 months | 40%-80% THR | MoCA  VO2 | 5/2 |
| Eich 2004 | T: 62.4 ± 4.8  C: 64.0 ± 6.0 | stork＜3 months | 17/33 | 25/25 | HIIT＋Routine exercises | Routine exercises | 60 min/times  5 times/week | 6 weeks | NA | 6WMT | 1/0 |
| El-Tamawy 2014 | T: 48.4 ± 6.39  C: 49.67 ± 6.98 | stork＞3 months | 21/9 | 30/21 | HIIT＋Routine exercises | Routine exercises | 25-30min/times  3 times/week | 8 weeks | NA | BNDF | NA |
| Fang 2003 | T: 65.49 ± 10.94  C: 61.8 ± 10.94 | stork＜3 months | 77/51 | 78/78 | MAT | Routine exercises | 45 min/times  5 times/week | 4 weeks | NA | MMSE | 28/0 |
| Hsu 2021 | T: 58.5 ± 12.89  C: 53.1 ± 10.22 | stork＞3 months | 20/3 | 10/13 | HIIT | MAT | 30-45min/times  6 times/month | 6 weeks | 60%VO_2_peak  OR  40%-80% VO_2_peak | MMSE  BNDF  VO2 | 3/2 |
| Ihle-Hansen 2019 | T: 71.4 ± 11.3  C: 72.0 ± 11.3 | stork＞3 months | 219/143 | 117/185 | HMAT＋Routine exercises | Routine exercises | 45-60min/times  2-3 times/week | 18 months | NA | MMSE | 34/29 |
| Koch 2020 | T: 59 ± 11  C: 58 ± 12 | stork＞3 months | 81/50 | 86/45 | HIIT＋Routine exercises | Routine exercises | 40-60min/times  3 times/week | 12 weeks | 50%-65% maximum heart rate | MoCA  6WMT | NA |
| Lapointe 2023 (a) | T: 71.8 ± 9.9  C: 69.6 ± 10.7 | stork＜3 months | 23/13 | 19/17 | HMAT | Routine exercises | 3 times/week | 12 weeks | 50%PPO  OR  30%-95%  PPO | MoCA  BMI  VO2 | 4/5 |
| Lapointe 2023 (b) | T: 65.6 ± 11.3  C: 69.6 ± 10.7 | stork＜3 months | 20/13 | 16/17 | MAT | Routine exercises | 3 times/week | 12 weeks | 50%PPO  OR  30%-95%  PPO | MoCA  BMI  VO2 | 3/5 |
| Munari 2018 | T: 60.87 ± 5.77  C: 61.71 ± 11.27 | stork＞3 month | NA | 8/7 | HIIT | LAT | 50-60min/times  3 times/week | 12 weeks | 85%-95% VO_2_peak  OR  40%-60%  VO_2_peak | 6WMT  BMI  VO2 | NA |
| Ploughman 2019 | T: 55 ± 7.41  C: 50 ± 8.89 | stork＞3 months | 14/16 | 16/14 | HIIT＋cognitive game | Routine exercises＋cognitive game | 40-45min/times  2 times/week | 4 weeks | 60%-80% VO_2_peak | VO2  BNDF | 3/5 |
| Quaney 2009 | T: 64.10 ± 12.30  C: 58.96 ± 14.68 | stork＞3 months | 17/21 | 19/19 | HIIT | Routine exercises | 45min/times  3 times/week | 8 weeks | 70% maximum heart rate | VO2 | NA |
| Vahlberg 2017 | T: 72.6 ± 5.5  C: 73.7 ± 5.3 | stork＞3 months | 51/16 | 34/33 | HIIT | Routine exercises | 75min/times  2 times/week | 3 months | NA | 6WMT | 8/2 |
| Liu 2022 (a) | 63.2 ± 8.3 | stork＞3 months | 127/53 | 30/30 | HIIT＋Routine exercises | LAT＋Routine exercises | 30min/times  5 times/week | 8 weeks | 50%-80% maximum heart rate | 6WMT | 0/0 |
| Liu 2022 (b) | 63.2 ± 8.3 | stork＜3 months | 127/53 | 30/30 | HIIT＋Routine exercises | LAT＋Routine exercises | 30min/times  5times/week | 8 weeks | 50%-80% maximum heart rate | 6WMT | 0/0 |
| Yeh 2022 | T: 53.05 ± 14.53  C: 60.17 ± 12.13 | stork＞3 months | 25/13 | 20/18 | HIIT＋cognitive game | Routine exercises | 45-60min/times  3 times/week | 12 weeks | 40%-70% maximum heart rate | MoCA | 0/0 |

**HIIT high-intensity interval training, HAT high-intensity aerobic training, MAT moderate-intensity aerobic training. HMAT high and moderate-intensity aerobic training; LAT low-intensity aerobic training; HRR heart rate reserve; THR target heart rate; VO_2_peak maximal oxygen uptake; PPO, peak power output; NA Not Applicable.**
