# Supplementary material for: The effect of moderate and vigorous aerobic exercise training on the cognitive and walking ability among stroke patients during different periods: A systematic review and meta-analysis
Source: PLoS One. 2024 Feb 23;19(2):e0298339. doi: 10.1371/journal.pone.0298339 (PMC10889575; doi:10.1371/journal.pone.0298339)
Supplement: S3 Table — (DOCX) [file pone.0298339.s003.docx]

**Table S3. The Result of PEDro Scale**

| **Study** | **PEDro** | | | | | | | | | | | **Sum PEDro** |
| --- | --- | --- | --- | --- | --- | --- | --- | --- | --- | --- | --- | --- |
|  | 1 | 2 | 3 | 4 | 5 | 6 | 7 | 8 | 9 | 10 | 11 |  |
| Boyne 2022 | 1 | 1 | 1 | 1 | 0 | 0 | 0 | 1 | 1 | 1 | 1 | 8 |
| Deijle 2022 | 1 | 1 | 1 | 1 | 0 | 0 | 1 | 1 | 1 | 1 | 1 | 9 |
| Eich 2004 | 1 | 1 | 1 | 1 | 0 | 0 | 1 | 1 | 1 | 1 | 1 | 9 |
| El-Tamawy 2014 | 1 | 0 | 0 | 1 | 0 | 0 | 0 | 1 | 1 | 1 | 1 | 6 |
| Fang 2003 | 1 | 1 | 1 | 1 | 0 | 0 | 0 | 1 | 1 | 1 | 1 | 8 |
| Hsu 2021 | 1 | 1 | 1 | 1 | 1 | 0 | 1 | 1 | 1 | 1 | 1 | 10 |
| Ihle-Hansen 2019 | 1 | 1 | 0 | 1 | 1 | 0 | 0 | 1 | 1 | 1 | 1 | 8 |
| Koch 2020 | 1 | 1 | 0 | 1 | 0 | 0 | 0 | 1 | 1 | 1 | 1 | 6 |
| Lapointe 2023 | 1 | 1 | 0 | 1 | 0 | 0 | 0 | 1 | 1 | 1 | 1 | 7 |
| Liu 2022 | 1 | 0 | 0 | 1 | 0 | 0 | 0 | 1 | 1 | 1 | 1 | 6 |
| Munari 2018 | 1 | 1 | 0 | 1 | 1 | 0 | 0 | 1 | 1 | 1 | 1 | 8 |
| Ploughman 2019 | 1 | 1 | 1 | 1 | 0 | 0 | 1 | 1 | 1 | 1 | 1 | 9 |
| Quaney 2009 | 1 | 1 | 1 | 1 | 0 | 0 | 0 | 1 | 1 | 1 | 1 | 8 |
| Vahlberg 2017 | 1 | 1 | 1 | 1 | 0 | 1 | 0 | 1 | 1 | 1 | 1 | 9 |
| Yeh 2022 | 1 | 1 | 1 | 1 | 0 | 0 | 1 | 1 | 1 | 1 | 1 | 9 |

**PEDro-scale-items: 1) Was eligibility criteria specified? 2) Were all subjects randomly allocated?3) Were allocations concealed?4) Were the groups similar at baseline? 5) Was there blinding of all participants? 6) Was there blinding of all therapists? 7) Was there blinding of all assessors? 8)Was there measures of at least one key outcome for more than 85% of the subjects initially allocated to groups? 9) Did all subjects for whom outcome measures were available receive the treatment or control condition as allocated or, where this was not the case, data for at least one key outcome was analyzed by “intention to treat”?10) Were the results of between group statistical comparisons reported for at least one key outcome? 11) Did the study have both point measures and measures of variability for at least one key outcome.**
