# Supplementary material for: The effect of moderate and vigorous aerobic exercise training on the cognitive and walking ability among stroke patients during different periods: A systematic review and meta-analysis
Source: PLoS One. 2024 Feb 23;19(2):e0298339. doi: 10.1371/journal.pone.0298339 (PMC10889575; doi:10.1371/journal.pone.0298339)
Supplement: S1 Checklist — (DOCX) [file pone.0298339.s004.docx]

| **Section** **and**  **Topic** | **Item** **#** | **Checklist** **item** | **Location** **where** **item** **is** **reported** |
| --- | --- | --- | --- |
| **TITLE** | | |  |
| Title | 1 | This report is a systematic review and Meta-analysis | 1 |
| **ABSTRACT** | | |  |
| Abstract | 2 | The abstract of this report includes the objective, method, results, and conclusion. | 2 |
| **INTRODUCTION** | | |  |
| Rationale | 3 | The current study lacks a review of the rehabilitation effects of aerobic exercise on patients in different stroke periods. | 3 |
| Objectives | 4 | To examine the effect of moderate and vigorous aerobic exercise training on the independent living ability of stroke patients. | 3 |
| **METHODS** | | |  |
| Eligibility criteria | 5 | We made the eligibility criteria to search relevant studies. | 4 |
| Information  sources | 6 | We used the Web of Science, Cochrane Library, Embase, CNKI databases, and PubMed for article retrieval. | 4 |
| Search strategy | 7 | Based on each database, different combinations of possible keywords and/or Medical Subject Headings terms were used. | 4 |
| Selection process | 8 | We chose the titles, abstracts, and full papers of pertinent studies using predetermined criteria. | 5 |
| Data collection  process | 9 | We extracted the following data from the included articles: study design, information of participants (age, gender, numbers), intervention design (experimental and control), treatment condition (frequency and duration), outcome measure, follow-up, and drop-out. Afterward, we created a common table by using these data | 5 |
| Data items | 10a | We used the Mini-Mental State Examination score (MMSE) and Montreal Cognitive Assessment (MoCA). we used 6-minute walk test(6WMT) to evaluate walking capacity. | 5 |
|  | 10b | The secondary outcomes are BNDF changes in VO_2_peak. | 5 |
| Study risk of bias assessment | 1 1 | We evaluated the risk of bias for each article using the Cochrane Handbook for Systematic Reviews of Interventions. To assess the caliber of the included research, we also employed the PEDro scale, which has 11 components | 6 |
| Effect measures | 12 | For outcome indicators, we use the form of mean ± standard deviation and a 95 % confidence interval. | 6 |
| Synthesis  methods | 13a | We grouped the data according to the outcome indicators of the experimental results, and grouped the data according to the type of intervention, intervention time, etc. | 6 |
|  | 13b | We make sure the unit of outcome is uniform. If the unit is not uniform, we will perform unit conversion when analyzing the data. | 6 |
|  | 13c | We extracted relevant information from the included literature and made it into a table. We make the outcome indicators into a forest map for analysis, and the results of the forest map analysis show the effectiveness of the data. | 6 |
|  | 13d | Using Review Manager Software, we conducted analyses on each of the papers we included (RevMan5.2). Because the data included in the study are continuous data, we pooled the data, chose the standardized mean difference (SMD) as a useful sign, and provided a 95% confidence interval (CI) for the variance. The I^2^ statistic was used to quantify heterogeneity, and the Cochrane Q statistic was used to determine whether heterogeneity occurred among the included studies (test level = 0.05). | 6 |
|  | 13e | If the level of heterogeneity is too high, we will do subgroup analysis and sensitivity analysis to identify the factors contributing to it. If it is impossible to pinpoint the exact cause of heterogeneity, a descriptive analysis is conducted. | 6 |
|  | 13f | We solved the problem of high heterogeneity of results through subgroup analysis and descriptive analysis, so we did not use sensitivity analysis to analyze. | not |
| Reporting bias  assessment | 14 | To assist the risk of bias, we evaluated the risk of bias for each included study by using the Cochrane Handbook for Systematic Reviews of Interventions and the PEDro scale. If two reviewers dispute the results of the study, consult a third reviewer for a decision. | 6 |
| Certainty  assessment | 15 | We used the P value and 95 % CI to describe the validity and feasibility of the results. | 6 |

| **Section** **and**  **Topic** | **Item** **#** | **Checklist** **item** | **Location** **where** **item** **is** **reported** |
| --- | --- | --- | --- |
| **RESULTS** | | |  |
| Study selection | 16a | We screened the literature according to the flow chart provided by PRISMA and drew the flow chart. | 6 |
|  | 16b | After reading the full text of the included studies, we excluded 6 articles, reasons being: Incomplete data (n = 3), Ineligible controls (n = 3). | 6 |
| Study  characteristics | 17 | A total of 15 articles were included in this study, of which 7 articles involved indicators related to cognitive ability (MMSE and MoCA), 8 articles applied 6WMT, 3 articles studied the numerical changes of BNDF and 6 articles measured the changes of VO_2_peak after exercise intervention. | 7 |
| Risk of bias in  studies | 18 | We used Table and Figure to show the result of Risk of bias in studies. | 7 |
| Results of individual studies | 19 | We show the results of the study on each outcome index by means of forest plots. | 8 |
| Results of  syntheses | 20a | We briefly summarise the characteristics and risk of bias among contributing studies. | 7 |
|  | 20b | The 95 % CI we provided for each of the results in the study | 8 |
|  | 20c | We did the results of all investigations of possible causes of heterogeneity among study results. | 8 |
|  | 20d | We solved the problem of high heterogeneity of results through subgroup analysis and descriptive analysis, so we did not use sensitivity analysis to analyze. | not |
| Reporting biases | 21 | We did assessments of the risk of bias due to missing results (arising from reporting biases) for each synthesis assessed. | 8 |
| Certainty of  evidence | 22 | We did assessments of certainty (or confidence) in the body of evidence for each outcome assessed by p-value. | 8 |
| **DISCUSSION** | | |  |
| Discussion | 23a | We provide a general interpretation of the results in the context of other evidence. | 9 |
|  | 23b | We discuss any limitations of the evidence included in the review. | 10 |
|  | 23c | We discuss any limitations of the review processes used. | 10 |
|  | 23d | We discuss the implications of the results for practice, policy, and future research. | 11 |
| **OTHER** **INFORMATION** | | |  |
| Registration and protocol | 24a | We provide registration information for the review. (PEOSPERO: CRD42023404448) | 4 |
|  | 24b | We write the plan on the relevant website of PEOSPERO and obtain the registration number. (PEOSPERO: CRD42023404448) | not |
|  | 24c | We did not make any amendments to information provided at registration or in the protocol. | not |
| Support | 25 | We did not describe sources of financial in the main document. | not |
| Competing  interests | 26 | We did not declare competing interests of review authors in the main document. | not |
| Availability of  data, code and  other materials | 27 | We provide access to all the studies included in this study. | 14 |

*From:* Page MJ, McKenzie JE, Bossuyt PM, Boutron I, Hoffmann TC, Mulrow CD, et al. The PRISMA 2020 statement: an updated guideline for reporting systematic reviews. BMJ 2021;372:n71. doi:

10. 1136/bmj.n71

For more information, visit:<http://www.prisma-statement.org/>
